# Synergism Between Water Management and Phosphorus Supply Enhances the Nodulation and Root Growth and Development of Chinese Milk Vetch (*Astragalus sinicus* L.)

**DOI:** 10.3389/fpls.2021.784251

**Published:** 2022-02-03

**Authors:** Zhengguo Sun, Mingxuan Yi, Xinbao Liu, Shen Yixin, Jianlong Li

**Affiliations:** ^1^College of Agro-Grassland Science, Nanjing Agricultural University, Nanjing, China; ^2^School of Life Sciences, Nanjing University, Nanjing, China

**Keywords:** Chinese milk vetch, soil moisture, phosphorus fertilizer application, interaction effect, root growth, root nodules nitrogen fixation

## Abstract

The response of root development and nodule formation of the manure crop Chinese milk vetch to different levels of soil moisture and phosphorous (P) fertilizer remains unclear. In this study, a pot experiment was performed to trace the root growth and nodule formation of Chinese milk vetch at the seedling, branching and full-flowering stages, under various soil moisture gradients [25% (W1), 50% (W2), 75% (W3), and 100% (W4) of the maximum field water-holding capacity] and P levels [0 (P0), 30 (P1), 60 (P2), and 90 (P3) kg hm^–2^]. The root/shoot ratio, root vitality, number of nodules, nodule weight, and nitrogenase activity were affected remarkably by soil moisture or the level of added P across the whole stage. Differences were found in the interaction effect between soil moisture and added P on the characteristic indices of the root and nodule at the different growth stages. There were obvious differences in root activity and nitrogenase activity at seedling stage, but no evident differences were found in other indices. Certain differences were also found in the indicators mentioned above at the branching stage. W1P0 and W2P0 showed the highest root/shoot ratio, W2P2 and W3P2 resulted in the highest root activity; W3P3 and W3P2 had the highest number and weight of nodules; and W3P2, W2P2, and W3P1 had higher nitrogenase activity than the other treatments at the full-flowering stage. The application of P at 60 kg hm^–2^ and the relative soil moisture of 75% was the best P-water combination suitable for the root development, nodule formation, and nitrogen fixation of Chinese milk vetch. This study will provide a theoretical basis for the production of this plant by managing the synergistic interaction between P fertilizer and soil moisture.

## Introduction

Chinese milk vetch (*Astragalus sinicus* L.), which is legume, is extensively cultivated in southern China and can be used as pasture, green manure, seasonal vegetables, and nectar plants (Sang-Yeol et al., 2008; Lee et al., 2015). In the 1970s and 1980s, the planting of this species was very popular, with an area of ∼7 × 10^6^ hm^2^. Chinese milk vetch is turned over and returned to the field in the middle and late stages of growth to increase the organic matter and other nutrients in the soil. This practice significantly enhances the yield of subsequent crop, rice (Aulakh et al., 2000; Cho et al., 2003). In recent years, China has implemented a crop rotation system and a double-reduction policy for chemical fertilizers and pesticides in some areas with greater ecological protection pressure or more serious continuous cropping obstacles. The country has also put forward an agricultural model of winter green manure crop–rice rotation to optimize the planting structure, adjust the stubble gap, and improve soil fertility of the cultivated land in southern regions, such as the Yangtze River Basin (Ma et al., 2020; Zhan et al., 2021). In the context of this policy, the planting of Chinese milk vetch has again risen substantially, with a sharp increase in the cultivated area.

Over the years, with the excessive pursuit of crop yields in agricultural production, chemical fertilizers and pesticides have been widely applied to farmlands, which has greatly damaged the ecological environment in such regions. In addition, long-term continuous cropping has seriously affected the physical and chemical properties of the soil, including soil compaction, low phosphorus (P) availability, and decreased soil water-holding capacity (Smith et al., 1997; Johnston et al., 2014; Roy et al., 2021a). Moreover, the soil microbiota has undergone major changes, such as the lack or poor vigor of rhizobial species that have a strong symbiotic relationship with the main green manure varieties (Liu et al., 2020). Chinese milk vetch, as an important green manure crop, is often exposed to various environmental conditions in the soil and is affected by multiple external factors, such as soil moisture and nutrient availability (Farrelly et al., 2011). Therefore, the effects of fertilizer and water conditions on green manure crops, especially under the conditions of rice rotation with abundant rainfall, should be considered to provide a certain theoretical support for efficient cultivation models and variety selection of green manure crops in the high-humidity areas in south China.

Plant growth and development are directly related to the nutrients and water absorbed by the roots from the soil. The Chinese milk vetch possesses a tap root system that penetrates deeply into the soil to obtain nutrients and water. The nodules formed on the lateral roots establish a unique symbiotic nitrogen (N) fixation system of legumes and play an essential role in the N fixation of Chinese milk vetch (Peoples et al., 2009). Thus, the growth of this plant is significantly affected by nodulation and root development. The symbiotic N fixation of legumes is affected by many factors, such as the species of rhizobia and hosts and the environmental conditions for plant growth (Pant et al., 2015; Schulte et al., 2021). Previous studies on the effects of environmental conditions on the formation, development, and N fixation of nodules of leguminous plants have mainly focused on single physical or chemical property in the soil (Kulkarni and Nautiyal, 2000).

Phosphorus (P) is an essential component of many coenzymes in plants and participates in many key physiological and biochemical processes in plants, such as the formation of cell nuclei, energy material metabolism, and genetic material synthesis (Divito and Sadras, 2014). P affects the carbon and N metabolism of plants, and elevated P application increases the size and number of legume nodules and enhances the symbiotic N fixation (Vance et al., 2003; Magadlela et al., 2015). In addition, soil moisture is an important factor affecting the N fixation of legume nodules, and suitable soil moisture can promote the formation of nodules (Deak et al., 2019). The root system, as the entrance of fertilizer and water from the soil to the plant, relies on the status of soil fertilizer and water for elongation and functional maintenance (Lynch and Brown, 2008). This system can also be affected by the growth status of the above-ground part and the formation and distribution of photosynthetic products. Several studies have proven that nutrient conditions, such as N and P levels, and soil moisture influence the root biomass and root/shoot ratio (Qiu et al., 2014). However, relatively few studies have focused on the interactive effects of soil P condition and water content on the root development and nodulation of legume plants.

This study was performed to elucidate the effects of P level and soil moisture and their interaction on the root development and nodulation of Chinese milk vetch. The optimal P-water combination for the root growth, nodule formation, and N fixation of the plant was also determined. Chinese milk vetch was exposed to different levels of soil moisture and P fertilizer to evaluate the growth dynamics of the root system and nodules at the main growth stages. The results will provide a theoretical basis for the exploration of the optimal combination of soil moisture and P fertilizer that is suitable to enhance the root growth and nodule formation of Chinese milk vetch and for the high-yield cultivation of this species and other leguminous green manure crops.

## Materials and Methods

### Plant Material, Rhizobium Strain, and Plant Growth Substrate

The cultivar of Chinese milk vetch used was “Xinzi 1” and was provided by the Xinyang Academy of Agricultural Sciences in Henan Province, China. The surface of the seeds were disinfected with 5% sodium hypochlorite for 8 min and then washed with sterile distilled water. The seeds were germinated in a growth chamber at a controlled condition for 5 days (day/night: 29°C/25°C, 16 h/8 h photoperiod at 250 μmol m^–2^s^–1^).

The rhizobium strain, *Mesorhizobium huakuii* (7653R), which was selected and applied on the basis of previous research in our laboratory (Liu et al., 2020), sourced from the Microbiology Laboratory of Nanjing Agricultural University and was maintained on yeast mannitol agar (YMA) (mannitol (10 g L^–1^), K_2_HPO_4_⋅3H_2_O (0.33 g L^–1^), K_2_HPO_4_ (0.25 g L^–1^), MgSO_4_ (0.1 g L^–1^), NaCl (0.1 g L^–1^), yeast extract (0.3 g L^–1^), and agar (15 g L^–1^)] at 4°C. The strain was grown in the YMA liquid medium at 28°C and 220 rpm for 2–4 days until the culture optical density at 600 nanometers (OD_600_) reached 0.8–1.0. The bacteria were then collected and washed twice with sterilized distilled water, and dissolved in sterilized distilled water as inoculants.

The plant growth substrate was a mixture of sieved yellow-brown soil and river sand (2/1, v/v), which had been autoclaved before carrying out cultivation. Organic matter of soil was determined according to the wet digestion method with potassium dichromate reagent (Jones, 2017). Total N content was measured with Kjeldahl method (Bremner and Mulvaney, 1982), and soil NH4^+^-N concentration was estimated by the method developed by Norman and Stucki (1981). Available phosphorus of soil was determined by sodium hydrogen carbonate solution-Mo-Sb anti spectrophotometric method (Olsen and Sommers, 1982). The content of soil available potassium was firstly extracted by neutral ammonium acetate solution and then determined by flame photometer method (Knudsen et al., 1982). Soil pH was determined at a soil-to-water ration of 1:2.5 (Bao, 2000). Water-holding capacity of soil was evaluated by the method of single-ring (Forster, 1998), which may be expressed by the water content when soil saturation and excess water has been exuded by gravity. The main physical and chemical properties of the growth substrate were as follows: organic matter content, 27.56 g kg^–1^; total N, 1.17 g kg^–1^, NH_4_^+^-N, 63.18 mg kg^–1^; available P, 11.82 mg kg^–1^; available potassium (K), 72.39 mg kg^–1^; pH, 6.95; and water-holding capacity, 19.11%. The N, P, and K fertilizers were applied as urea, calcium superphosphate, and potassium chloride, respectively. The growth substrate was initially applied with 37.5 kg N hm^–2^ and 30 kg K_2_O hm^–2^ to ensure the adequate supply of N and K nutrition.

### Experimental Set Up

The experiment was conducted at the Pailou Teaching and Research Base of Nanjing Agricultural University in Jiangsu Province, southern China (32°01′ N, 118°51′ E) on 20 September 2020. The germinated seeds were transplanted into pots (height, 12 cm; upper diameter, 23 cm; 6 seedlings per pot) containing 3 kg of plant growth substrate. When the first leaf was fully expanded, 5 mL of rhizobium solution, which had been cultured on the medium, was inoculated into the roots, with sterile water as the blank control in the treatment of non-inoculated bacteria solution.

The experiment was arranged in a 4 × 4 factorial design, in which the main effects were the P levels (P_2_O_5_; no P added (P0, 0 kg⋅hm^–2^), low P (P1, 30 kg⋅hm^–2^), moderate P (P2, 60 kg⋅hm^–2^), and high P (P3, 90 kg⋅hm^–2^)] and soil moisture [low water condition (W1, 25% water-holding capacity), moderate water (W2, 50%), suitable water (W3, 75%), and high water (W4, 100%)]. The application of three different phosphorus concentrations mentioned above was mainly based on the production practice of winter crops, especially legume green manure crops in the middle and lower reaches of the Yangtze River. Nine replicate pots were conducted for each treatment. The pots were randomly placed in a greenhouse.

The soil moisture content was controlled by the weighing method at the 5-leaf stage of the Chinese milk vetch. The pots were weighed at 16:00–17:00 and were added with water according to the four moisture gradients. Considering that the pot was in a natural environment involving soil evaporation, upper and lower limits of the water gradient were set for each treatment. When the weight of the pot was lower than the lower limit, water was supplemented to the upper limit to ensure the moisture gradients.

### Sample Collection

Plant samples were collected during the seedling stage (20 December 2020), branching stage (15 February 2021), and full-blooming stage (30 March 2021). After rinsing, the roots were transported back to the laboratory in an ice box for further analysis.

### Calculation of the Root/Shoot Ratio and Nodule Properties

The root/shoot ratio indicated the ratio of the dry weight of the root and the above-ground part of the plant. The number of nodules on the root was counted. The individual nodule biomass was calculated from the data of the nodule fresh weight per plant.

### Determination of Root Vitality and Nitrogenase Activity

Root vitality generally referred to the ability of roots to absorb and transport water and nutrients, and to synthesize and transport important organic matter, which was usually determined by triphenyltetrazolium chloride (TTC) reduction method. Briefly, the amount of TTC reduced to triphenylformaldehyde in the roots was analyzed as an indicator of root vitality. Absorbances were recorded at 520 nm (Clemensson-Lindell, 1994). Nitrogenase activity was determined via the acetylene reduction method as previously reported (Liu et al., 2020) and was expressed as ethylene produced per gram of fresh nodule per minute.

### Statistical Analyses

The experimental data were analyzed using Microsoft Office Excel (Excel 2016) and SPSS analytical software (SPSS 23.0). The experimental data were obtained from at least three biological replicates. The results were expressed as mean ± standard error (SE). Most data were subjected to a two-way ANOVA method with P levels and water status as the factors, followed by Duncan’s test to determine significant differences (*P* < *0.05*).

## Results

### Effects of P Levels and Soil Moisture on the Root/Shoot Ratio of Chinese Milk Vetch

**FIGURE 1:**
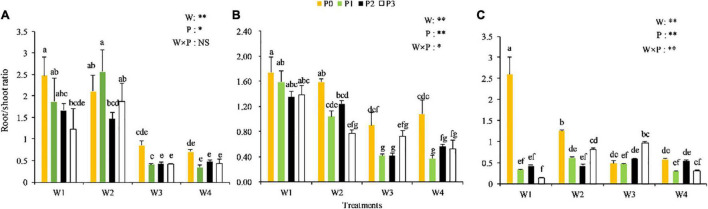
Root/shoot ratio of Chinese milk vetch (CMV) at the different soil moisture contents and levels of added phosphorus. **(A–C)** Represents the seedling stage, branching stage, and full-flowering stage, respectively. In the legend, W represents the effect of soil moisture, P represents the influence effect of phosphorus application rate change, and W × P represents the interaction between soil moisture and P application. *Means the difference is significant at the 0.05 level, and **Means the difference is significant at the 0.01 level. The lowercase letter above the column in the bar chart have no significant difference for the same ones, while there are significant differences for different ones. The same as Figures 2, 3.

The root/shoot ratio of Chinese milk vetch underwent certain changes in response to the various combinations of P levels and soil moisture at different growth stages (Figure 1). The root/shoot ratio generally decreased from the seedling to the full-flowering stage. At each stage, the root/shoot ratio first increased and then decreased with the increase in soil water content. In addition, as the P levels increased, the root/shoot ratio decreased to a certain extent. No significant P × water interaction on the root/shoot ratio was observed at the seedling stage, but the influence of soil moisture was greater than that of the P levels. At the branching stage, the root/shoot ratio was significantly affected by the interaction between the P levels and soil moisture (*P* < 0.05). A significant P × water interaction was also observed at the full-blooming stage (*P* < 0.05), with relatively higher root/shoot ratios observed in the W1P0, W2P0, and W3P3 treatments compared with the other treatments. This phenomenon indicated that low P and low water conditions might be beneficial in maintaining a high root/shoot ratio. Thus, increasing the application of P fertilizer under suitable soil moisture condition may increase the root/shoot ratio, but high soil moisture could significantly reduce this index.

### Effects of P Levels and Soil Moisture on the Root Vitality of Chinese Milk Vetch

**FIGURE 2:**
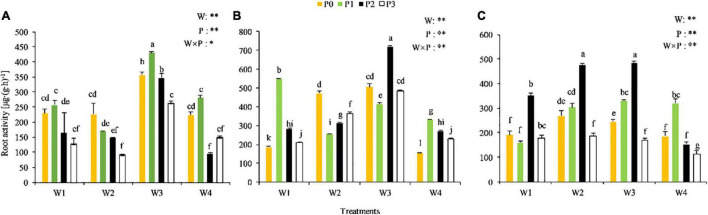
Root activity of CMV at the different soil moisture contents and levels of added phosphorus. **(A–C)** Represents the seedling stage, branching stage, and full-flowering stage, respectively.

The root vitality varied among treatments at the different growth stages (Figure 2). A significant P × water interaction was observed at the seedling stage (*P* < 0.05), while highly significant P × water interactions were observed at the branching and full-blooming stages (*P* < 0.01). The root vigor of Chinese milk vetch was relatively higher in the W3P1, W3P2, and W3P0 treatments at the seedling stage, in the W3P2 and W1P1 treatments at the branching stage, and in the W3P2 and W2P2 treatments at the full-blooming stage, compared with the other treatments. The root vitality performed well in the W3P2 treatment across the growth stages, and the value at seedling stage, branching stage and full-blooming stage was 346.04, 719.77, and 483.22 μg⋅(g⋅h)^–1^, respectively.

### Effects of P Levels and Soil Moisture on the Nodulation of Chinese Milk Vetch

**FIGURE 3:**
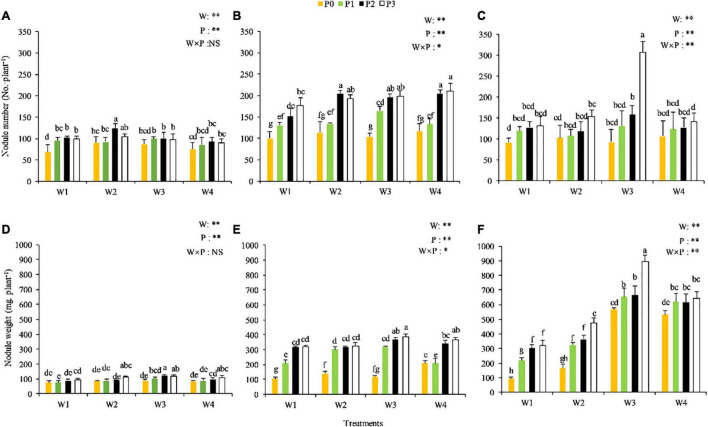
Number and weight of nodules of the CMV at the different soil moisture contents and levels of added phosphorus. **(A,D)** Represent the seedling stage, **(B,E)** represent branching stage, and **(C,F)** indicate as full-flowering stage.

No significant P × water interaction on the number of nodules was observed at the seedling stage (*P* > 0.05) (Figure 3A), but significant P × water interactions were observed at the branching and full-blooming stages (*P* < 0.05) (Figures 3B,C). Compared with other treatments, the numbers of nodules were relatively higher in the W2P2, W2P3, W1P2, and W3P2 treatments at the seedling stage, in W4P2, W4P3, W2P2, W3P3, W3P2, and W2P3 treatments at the branching stage, and in W3P3 treatment at the full-blooming stage. The plant in the W1P0 treatment displayed the lowest number of nodules across the three growth stages, indicating that drought and P deficiency might limit the nodule formation of Chinese milk vetch.

The fresh weight of the nodule was significantly influenced by the interaction between the P level and soil moisture and increased with the progression of growth stage (Figures 3E,F). No significant P × water interaction on the fresh weight of the nodule was observed at the seedling stage (*P* > 0.05) (Figure 3D). However, highly significant P × water interaction was observed at the branching stage with W3P2, W3P3, and W4P3 treatments showing the highest fresh weight (over 360 mg per plant) (*P* < 0.01), and at the full-blooming stage with W3P3 treatment showing the highest fresh weight (over 900 mg per plant), and with W3P2, W3P1, W4P3, W4P1, and W4P2 treatments showing relatively high fresh weight (over 600 mg per plant) (*P* < 0.01). These results indicated that adequate water facilitated the growth and development of nodules.

### Effects of P Levels and Soil Moisture on the Nitrogenase Activity of Chinese Milk Vetch

The main effect of soil moisture and P level on the nitrogenase activity of the Chinese milk vetch root nodule significantly differed in each main growth period (Table 1, *P* < 0.05). When the amount of P applied was constant, the nitrogenase activities in the W3 treatments were the highest across the three stages. Compared with the nitrogenase activity under the W3 treatment, the nitrogenase activity under other moisture conditions decreased by 35.81–73.17%, 24.59–47.86%, and 14.74–77.95% at the seedling, branching and full-blooming stages, respectively. When the soil moisture was constant, the nitrogenase activity at the seedling, branching, and blooming stages increased first and then decreased with the increase in the amount of P applied. These activities reached maximum values at P2 application and then decreased significantly (*P* < 0.05), among which the drop during the seedling stage was the largest (76.12%).

**TABLE 1:** Nitrogenase activity of Chinese milk vetch (CMV) under different soil moisture contents or levels of added phosphorus.

Effect factor	Treatment	Nitrogenase activity (nmol C_2_H_4_⋅min^–1^⋅g^–1^)		
		Seedling stage	Branching stage	Full-flowering stage
Soil moisture (%)	W1	2.42 ± 0.33^d^	49.13 ± 2.60^d^	51.74 ± 5.70^c^
	W2	4.95 ± 0.68^c^	59.04 ± 4.21^c^	200.07 ± 18.99^b^
	W3	9.02 ± 1.39^a^	94.22 ± 7.43^a^	234.66 ± 21.47^a^
	W4	5.79 ± 1.05^b^	71.05 ± 5.91^b^	193.10 ± 14.45^b^
Phosphorus application	P0	4.06 ± 0.77^c^	53.82 ± 4.77^c^	137.34 ± 19.08^c^
(kg⋅hm^–2^)	P1	6.54 ± 0.77^b^	74.78 ± 5.88^b^	180.92 ± 27.37^b^
	P2	9.34 ± 1.31^a^	90.24 ± 8.11^a^	222.89 ± 32.82^a^
	P3	2.23 ± 0.34^d^	54.61 ± 3.28^c^	138.41 ± 14.20^c^

*The lowercase letters in the table represent whether there is significant difference. The same letters represent insignificant differences, while different letters represent significant differences. The same as Table 2.*

A highly significant P × water interaction on the nitrogenase activity of Chinese milk vetch was observed in this study (Table 2, *P* < 0.01). Under constant P levels, compared with that under W1 treatment, the nitrogenase activity under the W3 treatment increased as follows: 256.96, 211.04, 338.81, and 264.29% at the P0, P1, P2, and P3 levels, respectively, at the seedling stage; 101.85, 85.74, 121.41, and 52.84% at the P0, P1, P2, and P3 levels, respectively, at the branching stage; and 408.99, 450.61, 394.46, and 193.93% at the P0, P1, P2, and P3 levels, respectively, at the full-blooming stage. These results suggested that increasing the soil moisture content within a certain range would promote the nitrogenase activity after a constant P supply. In addition, under constant soil moisture condition, the nitrogenase activity of the root nodule increased with the P supply, reached its peak at the P3 level, and then decreased at the P4 level. The lower nitrogenase activity in the W4P3 treatment compared with that in W4P0 indicated that, under a waterlogged condition, a negative interaction would occur between the P level and soil moisture on the nitrogenase activity of Chinese milk vetch, even if the P level was high.

**TABLE 2:** Response of nitrogenase activity of CMV to the synthesized effects of different soil moisture contents and levels of added phosphorus.

Treatments	Nitrogenase activity (nmol C_2_H_4_⋅min^–1^⋅g^–1^)		
	Seedling stage	Branching stage	Full-flowering stage
W1P0	2.37 ± 0.17^f^	36.75 ± 0.99^i^	34.12 ± 11.23^c^
W1P1	3.08 ± 0.25^f^	54.54 ± 0.40^efgh^	48.61 ± 11.42^c^
W1P2	3.53 ± 0.22^f^	59.12 ± 1.30^defg^	65.91 ± 8.39^c^
W1P3	0.70 ± 0.07^g^	46.12 ± 0.63^ghi^	58.33 ± 6.77^e^
W2P0	3.12 ± 0.25^f^	42.33 ± 0.56^hi^	172.95 ± 14.23^cd^
W2P1	5.54 ± 0.70^e^	67.11 ± 1.23^cde^	181.20 ± 31.00^cd^
W2P2	8.09 ± 0.43^d^	75.95 ± 1.63^c^	294.48 ± 15.68^a^
W2P3	3.03 ± 0.83^f^	50.77 ± 5.99^fgh^	151.65 ± 18.66^d^
W3P0	8.46 ± 0.15^c*d*^	74.18 ± 2.86^c^	173.67 ± 13.10^cd^
W3P1	9.58 ± 0.26^b*c*^	101.30 ± 2.45^b^	267.65 ± 18.33^ab^
W3P2	15.49 ± 0.47^a^	130.90 ± 1.64^a^	325.86 ± 25.62^a^
W3P3	2.55 ± 0.21^f^	70.49 ± 4.32^cd^	171.45 ± 20.48^cd^
W4P0	2.63 ± 0.08^f^	62.00 ± 6.25^cdef^	168.63 ± 20.05^cd^
W4P1	7.96 ± 0.63^d^	76.15 ± 12.89^c^	226.20 ± 38.94^bc^
W4P2	10.24 ± 0.60^b^	94.97 ± 4.02^b^	212.89 ± 34.84^bcd^
W4P3	2.31 ± 0.49^f^	51.05 ± 2.39^fgh^	164.65 ± 3.90^cd^
W	**	**	**
P	**	**	**
W × P	**	**	**

**Means the difference is significant at the level 0.01.

## Discussion

Chinese milk vetch is mainly used as a green manure incorporated into the soil. The amount of biomass of Chinese milk vetch directly determines the impact of Chinese milk vetch on the physical and chemical properties of the soil after being incorporated (Cho and Widholm, 2002; Roy et al., 2021b). The biomass of the root stubble also influences the bulk density, porosity, organic matter content, and the status of the rhizosphere microorganisms in the soil (Asagi and Ueno, 2009). Soil moisture and nutrients are directly or indirectly involved in various processes of the growth and development of plants (Roy et al., 2020; Corvalán Videla et al., 2021). In this research, we found that the growth and development of roots and nodule of Chinese milk vetch were greatly affected by insufficient or relatively more or even saturated water. The P fertilizer is particularly important for legumes (Kachiguma et al., 2019). Application of P can promote the root growth and nodule formation and increase the nitrogenase activity by facilitating the establishment of a symbiotic association with the soil rhizobium to fix more free N (Vardien et al., 2014). In this research, with the increase of phosphorus application in a certain range, the nitrogenase activity of nodule was significantly increased, which has been demonstrated in the three growth periods mentioned in this article. Therefore, the interaction between soil moisture and P fertilizer is directly related to the biomass and yield of Chinese milk vetch via the regulation of root development and nodule formation. In this study, we monitored the response of plant growth, nodule formation, and nitrogenase activity of Chinese milk vetch to the interaction between multiple P levels and soil moisture contents across the main growth stages. We found that a suitable soil moisture combined with a moderate P level was beneficial in the production of Chinese milk vetch.

Plants primarily rely on the root system to obtain water and mineral nutrients. Therefore, the growth and activity of the roots directly affect the development of the entire plant (Wain, 1977). According to the “functional equilibrium hypothesis” proposed by Brouwer (1962), plants are divided into two parts, namely underground and above ground. The growth of the underground part mainly depends on the absorption and utilization of water and nutrients by roots. The growth rate of the above-ground part is mostly limited by the carbon supply rate of photosynthesis. The root/shoot ratio, symbolizing the correlation between the development of the above-ground and the underground parts, could also be affected by soil moisture and mineral nutrition. Therefore, in this study, the root-shoot ratio of Chinese milk vetch at different growth stages changed with the change of phosphorus application rate and soil water condition. When the supply of P and water in the soil is insufficient, photosynthetic products will be preferentially distributed to the roots to ensure the normal growth of the plant root system. Nevertheless, when the supply of P and water is sufficient, the priority of nutrients is given to the above-ground part to increase the stems and leaves and to promote photosynthesis (Huang and Fry, 1998; Dodd and Diatloff, 2016). In this study, within a certain range, the efficiency of P utilization of the Chinese milk vetch increased as the soil moisture content gradually increased. This phenomenon might be related to the fact that soil moisture promoted root development and enhanced the ability of roots to obtain nutrients. In addition, the root vitality of Chinese milk vetch was also related to the degree of P-water treatment. Root activity was relatively high under treatments of appropriate water with low P (W3P1) and appropriate water with moderate P (W3P2) treatments, while waterlogging (W4) and high P level (P3) resulted in decreased root vitality.

The process of symbiotic N fixation of legumes is complex, which is mainly divided into two steps, namely, nodulation and N fixation. Shi et al. (2021) has reported that P application is an effective method to increase the number of effective nodules and improve the efficiency of N fixation of legumes. In this study, the number and fresh weight of nodule increased after the elevated P supply, indicating that P promoted the formation and growth of nodules. Nitrogenase activity plays an important role during the formation of legume nodules (Oldroyd and Allan, 2008; Mantovani et al., 2015). Nitrogenase is the main catalyst priming the N fixation in the legume–rhizobia symbiotic interaction. In the process of fixing free N into ammonia (NH_4_^+^-N), the energy required is generally provided by ATP (Tang et al., 2001), while P element is a key component of ATP. Thus, P plays a key role in the nodulation and N fixation of legumes. Deficiency in P will lead to the reduction of leghemoglobin content in the nodules, which in turn suppresses the nitrogenase activity (Kleinert et al., 2017; Htoon et al., 2019). Herein, the nitrogenase activity increased first and then decreased with the increase of P levels. High P condition inhibited nitrogenase activity, indicating that a critical range of P might exist for the nodule N fixation of the Chinese milk vetch. Increased P promoted N fixation within a lower concentration range but inhibited N fixation at higher concentrations. Moreover, these results suggest that soil moisture was closely related to nitrogenase activity. The nitrogenase activity of the nodules significantly decreased under drought conditions (W1) when compared to the highest nitrogenase activity under relatively higher water supply (W3). Chiozzotto et al. (2018) reported that low soil moisture reduced legume nitrogenase activity caused by the repressed absorption of fertilizers. Thus, a relatively higher soil moisture combined with a moderate P level (W3P2) contributed to the highest nitrogenase activity, which had been shown in the results section. This may be due to the synergistic effect of phosphorus and water on root growth and nodule formation by stimulating the interaction between root and rhizobium. Thus, the interaction between appropriate water management and P input plays an important role in the nodule development and the maintenance of high nitrogenase activity of Chinese milk vetch.

## Conclusion

The P level significantly affected the root/shoot ratio, number of nodules, fresh weight of the nodule, and nitrogenase activity of Chinese milk vetch. The nitrogenase activity increased and later decreased with elevated P supply. A suitable soil moisture facilitated the N fixation of the nodules, whereas drought repressed the growth and function of the nodules. A significant interaction was found between the P level and soil moisture. Our results suggested that a suitable soil moisture combined with a moderate P level, but not high P supply (W3P2 treatment in this study), was the optimal condition for root growth and nodule formation of Chinese milk vetch. Therefore, a reasonable management of soil moisture and P application is necessary for the efficient nodule formation and enhanced growth of Chinese milk vetch.

## Data Availability Statement

The original contributions presented in the study are included in the article/supplementary material, further inquiries can be directed to the corresponding author/s.

## Author Contributions

ZS and SY conceived the ideas. MY and XL collected the data. ZS and MY analyzed the dada and led the writing. XL, SY and JL provided more argumentations in the results and conclusion. ZS, SY and JL proofed the effectivity and rationality of the method proposed in this manuscript. All authors contributed critically to the ideas and drafts and gave final approval for publication.

## Conflict of Interest

The authors declare that the research was conducted in the absence of any commercial or financial relationships that could be construed as a potential conflict of interest.

## Publisher’s Note

All claims expressed in this article are solely those of the authors and do not necessarily represent those of their affiliated organizations, or those of the publisher, the editors and the reviewers. Any product that may be evaluated in this article, or claim that may be made by its manufacturer, is not guaranteed or endorsed by the publisher.
